# Hemato-CHEESE-ia: A Case of Red Stools Caused by a Spicy Cheesy Snack

**DOI:** 10.7759/cureus.28584

**Published:** 2022-08-30

**Authors:** Katie F Lee, Jared Magee, John McCarthy

**Affiliations:** 1 School of Medicine, Uniformed Services University of the Health Sciences, Bethesda, USA; 2 Department of Gastroenterology and Hepatology, Walter Reed National Military Medical Center, Bethesda, USA

**Keywords:** bloody stool, abdominal pain, capsaicin, red dye, hematochezia

## Abstract

A 22-year-old male presented to the ED following an episode of acute-onset abdominal pain and passage of red stools. On a detailed review of his dietary history, the patient reported eating a whole large bag of Flamin’ Hot Cheetos the day prior to the episode. We describe a case of food dye mimicking hematochezia and explore the effects of capsaicin on the gastrointestinal (GI) tract.

## Introduction

Hematochezia, or the passage of bright red blood per rectum, usually indicates lower gastrointestinal (GI) bleeding. The differential for a lower GI bleed is broad and ranges from benign entities to life-threatening conditions. Population-based studies have reported the incidence rate of acute GI bleeding to be 90-108 per 100,000 persons [1]. Acute GI bleeding results in 300,000 hospitalizations annually and hence warrants a robust approach to diagnosis and management. The first step is a primary assessment including dietary history, often followed by an endoscopic evaluation to further characterize the source of bleeding [2]. We discuss a case of a young male who presented with bright red stools secondary to consuming Flamin’ Hot Cheetos, highlighting the importance of including a thorough dietary history in any primary assessment for reported hematochezia.

This case report was previously presented as a poster at the 2022 Research & Innovation Month with The Department of Research Programs at the Walter Reed National Military Medical Center in Bethesda, MD on May 20, 2022.

## Case presentation

A 22-year-old male presented to the ED after an episode of acute-onset abdominal pain and one bowel movement that was concerning due to the appearance of blood. The patient reported that he had woken up in the middle of the night with moderate, diffuse abdominal pain and then had a bowel movement with a large amount of bright red stool. Earlier in the night, he reported that he had woken up with abdominal pain and had a non-bloody bowel movement that consisted of loose stool. At the time of the ED visit the next morning, his pain had subsided. He denied all other constitutional symptoms. His physical exam, which included a rectal exam, and laboratory testing (hemoglobin, hematocrit, erythrocyte sedimentation rate, C-reactive protein, and lipase) were found to be unremarkable. Given reassuring vital signs and resolution of symptoms, the patient was discharged from the ED with a follow-up scheduled for the next day with outpatient gastroenterology.

**Figure 1:**
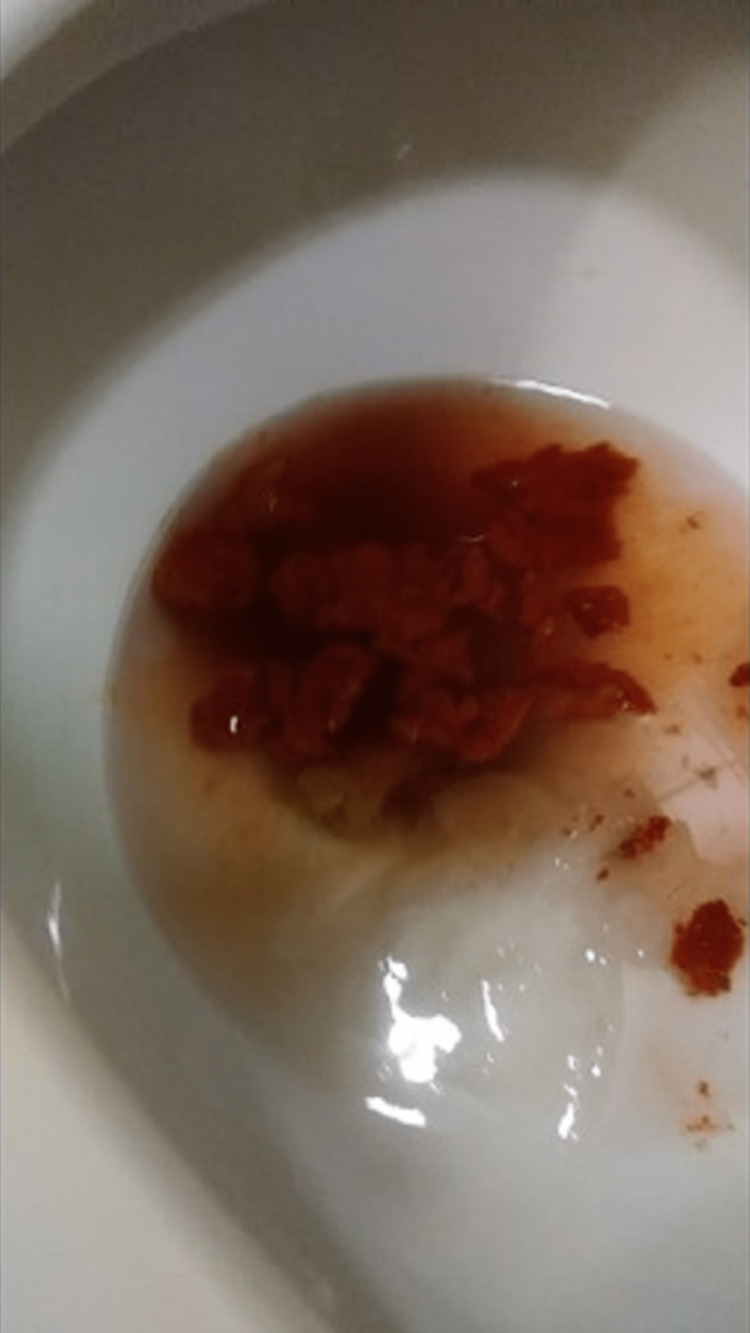
One episode of "bloody" stool recorded by the patient

Upon further questioning in the gastroenterology clinic, the patient characterized the stool as having a red/orange color. A picture he had taken and provided of this stool confirmed this to be an accurate description (Figure 1). He additionally endorsed that his abdominal pain was relieved after the bowel movement. On a detailed review of dietary history, the patient reported eating a whole large bag of Flamin' Hot Cheetos the day prior to the episode. The patient endorsed that it was a new dietary item for him. He denied having any additional bowel movements or abdominal pain since the episode, and his review of systems remained negative. The abdominal exam and digital rectal exam were repeated and found to be unrevealing. The patient was offered an endoscopic evaluation for further reassurance, but he ultimately agreed with the dietary etiology of his symptoms and was counseled on return precautions.

## Discussion

Algorithmic approaches to hematochezia often overlook the importance of taking a detailed dietary history. After resuscitation or clinical stability is established, initial assessments are often geared toward investigating the source of bleeding. This case report emphasizes the importance of taking a detailed dietary history during the initial assessment.

Several cases of red-colored stool caused by food or medication have been documented. Colored gelatin, candy, beets, rifampin, phenytoin, and cefdinir with Pedialyte have been cited as possible mimickers of bright red blood per rectum [3,4]. In another case of red-colored stool after colored gelatin ingestion, Red Lake 40 food coloring was likely the source [5]. The same dye is used in Flamin’ Hot Cheetos.

In a case of suspected GI bleeding, concurrent abdominal pain causes suspicion for specific etiologies, such as peptic ulcer disease (PUD) or ischemic colitis. However, in this case of food dye mimicking blood, the specific food ingested was likely also responsible for the patient's abdominal pain. Spicy foods often contain a chemical called capsaicin to supplement taste. When consumed in copious amounts, capsaicin can cause abdominal pain. Capsaicin, a vanilloid compound, induces the sensation of pain and heat in the GI tract via nociceptors. The chemical interacts with a specific receptor called transient receptor potential vanilloid 1 (TRPV1) on nociceptive neurons [6]. The mechanism of action entails the activation and then desensitization of TRPV1 receptors on nociceptive neurons. Topical forms of capsaicin utilize the desensitization aspect for pain relief [7]. In the GI tract, however, infusions of capsaicin have been shown to increase pain and burning in the duodenum and jejunum, as well as increased sensitivity to balloon distention in the stomach and ileum [6].

## Conclusions

In a patient with abdominal pain presenting with a concern for bloody stools, a broad differential should be considered, paying close attention to dietary history. This is especially true in healthy, young patients who do not have any predisposing medical conditions, take high-risk medications, or exhibit signs of hemorrhage. In the evaluation of rectal bleeding, the consideration that the presentation is due to a red substance other than blood should be made. The evaluation of this patient whose dietary history revealed prior consumption of a large bag of Flamin’ Hot Cheetos also led to the exploration of capsaicin eliciting abdominal pain and provided a unifying diagnosis of an unusual presentation.

## References

[1] Prasad Kerlin M, Tokar JL (2013). Acute gastrointestinal bleeding. Ann Intern Med.

[2] Strate LL, Gralnek IM (2016). ACG clinical guideline: management of patients with acute lower gastrointestinal bleeding. Am J Gastroenterol.

[3] Singhi S, Jain P, Jayashree M, Lal S (2013). Approach to a child with upper gastrointestinal bleeding. Indian J Pediatr.

[4] Eljaaly K, Alshehri S (2020). Cefdinir-induced red stool and purple discoloration of nutritional formula: a case report. J Infect Chemother.

[5] Sullivan SN (1993). Red jello stool and red dye-arrhea. J Clin Gastroenterol.

[6] Hammer J, Vogelsang H (2007). Characterization of sensations induced by capsaicin in the upper gastrointestinal tract. Neurogastroenterol Motil.

[7] Sharma SK, Vij AS, Sharma M (2013). Mechanisms and clinical uses of capsaicin. Eur J Pharmacol.

